# Concurrent behavioral and electrophysiological longitudinal recordings for *in vivo* assessment of aging

**DOI:** 10.3389/fnagi.2022.952101

**Published:** 2023-01-18

**Authors:** Christopher Daniel Morrone, Arielle A. Tsang, Sarah M. Giorshev, Emily E. Craig, Wai Haung Yu

**Affiliations:** ^1^Brain Health Imaging Centre, Centre for Addiction and Mental Health, Toronto, ON, Canada; ^2^Department of Biological Sciences, University of Toronto Scarborough, Toronto, ON, Canada; ^3^Department of Psychology, University of Toronto Scarborough, Toronto, ON, Canada; ^4^Geriatric Mental Health Research Services, Centre for Addiction and Mental Health, Toronto, ON, Canada; ^5^Department of Pharmacology and Toxicology, University of Toronto, Toronto, ON, Canada

**Keywords:** electroencephalogram (EEG), mouse behavior, PhenoTyper, sleep, Barnes maze, wireless, aging

## Abstract

Electrophysiological and behavioral alterations, including sleep and cognitive impairments, are critical components of age-related decline and neurodegenerative diseases. In preclinical investigation, many refined techniques are employed to probe these phenotypes, but they are often conducted separately. Herein, we provide a protocol for one-time surgical implantation of EMG wires in the nuchal muscle and a skull-surface EEG headcap in mice, capable of 9-to-12-month recording longevity. All data acquisitions are wireless, making them compatible with simultaneous EEG recording coupled to multiple behavioral tasks, as we demonstrate with locomotion/sleep staging during home-cage video assessments, cognitive testing in the Barnes maze, and sleep disruption. Time-course EEG and EMG data can be accurately mapped to the behavioral phenotype and synchronized with neuronal frequencies for movement and the location to target in the Barnes maze. We discuss critical steps for optimizing headcap surgery and alternative approaches, including increasing the number of EEG channels or utilizing depth electrodes with the system. Combining electrophysiological and behavioral measurements in preclinical models of aging and neurodegeneration has great potential for improving mechanistic and therapeutic assessments and determining early markers of brain disorders.

## 1. Introduction

Electrophysiological changes and disruptions in brain networking are associated with age-related cognitive decline and the development of neurodegenerative diseases (López-Sanz et al., 2019; McMackin et al., 2019; Saxena and Liebscher, 2020). Oscillatory signals captured by electroencephalography (EEG) correlate with behavioral deficits in patients with dementia and Alzheimer’s disease (AD), and in animal models, and include alterations in neuronal frequency activity and power, cross-frequency coupling, and lagged-phase synchronization (Jeong, 2004; Canolty and Knight, 2010; Hamm et al., 2015; Hata et al., 2016). Impairments in EEG signals can even be observed in people with mild cognitive impairment, a precursor to dementias like Alzheimer’s disease, making this a potentially useful tool for the early detection of dementia and neurodegeneration where subtle perturbations precede canonical disease neuropathology (Nakamura et al., 2018; Gaubert et al., 2019; van der Zande et al., 2020; Meghdadi et al., 2021). Despite the mounting evidence of neuronal network dysfunction affecting learning and memory in neurological and psychiatric disorders, preclinical techniques for longitudinal *in vivo* electrophysiological and behavioral assessments in freely moving animals are under utilized. This limits the ability to identify multimodal, synchronous changes such as electrophysiology and behavior affected by age and disease progression.

One such prominent contributor to, and the outcome of, neuronal network dysfunction is sleep abnormalities, which have long been known to occur (∼50–60 years) in brain disorders (Ginzberg, 1955; Kupfer and Foster, 1972; Wang et al., 2015). Normal aging is associated with sleep disturbances in the daily sleep–wake cycle; however, the sleep loss associated with AD appears to be an exacerbation of these changes (Vitiello and Borson, 2001). Dementias are associated with hyperarousal states, including increased night-time awakenings, decreased rapid eye movement (REM), and non-REM (NREM) sleep, in particular, delta-wave-dominant slow wave sleep or NREM stage 3 (Vitiello et al., 1991; Vitiello and Borson, 2001; Carter et al., 2010). These stages are normally critical for the cognitive and restorative benefits of sleep (Walker and Stickgold, 2004; Brown et al., 2012; Tononi and Cirelli, 2014). In fact, over 60% of patients with mild cognitive impairment or dementia report sleep disruptions, including a ∼45–50% prevalence of insomnia (Guarnieri et al., 2012). The presence of sleep impairments confers a high risk for mild cognitive impairment and AD, notably in the development of AD biomarkers, 3.78× (Bubu et al., 2017), adding to the importance of investigating early EEG changes as it relates to behavioral changes. The correlative relationship between sleep and neurodegenerative disease may reciprocally drive disease progression, in which loss of sleep can exacerbate neurodegenerative processes, and the accumulation of proteinopathy can contribute to sleep impairments (Musiek and Holtzman, 2016; Minakawa et al., 2019). Critically, sleep disruption and hyperarousal are associated with aging and contribute to impaired cognitive performance in an array of neurodegenerative (i.e., AD, frontotemporal dementia, and Parkinson’s disease) and psychiatric disorders (i.e., depression and schizophrenia) (Liu et al., 2004; Ju et al., 2014; Chahine et al., 2017; Fang et al., 2019; Waite et al., 2020; Wainberg et al., 2021).

**FIGURE 1:**
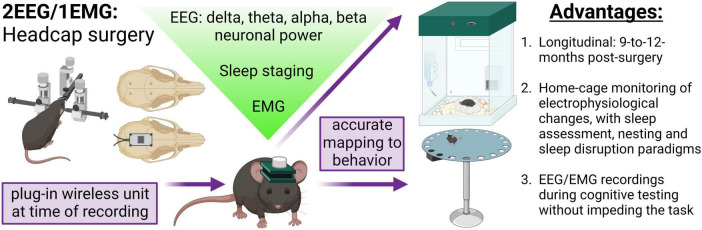
Concurrent EEG/EMG recordings with behavioral testing in mice. Schematic of the method employed. Mice underwent stereotaxic surgery to implant a headcap with two EEG channels and EMG wires in the nuchal muscle. EEG signal was derived from four stainless steel screw electrodes implanted at Bregma –2–2.5 mm AP, 1.5 ML, and Bregma 3.5–4 mm AP, 1.5 ML, as approximately indicated by the red × markings. A lightweight and small headcap was connected to the screws on the skull top and secured with dental cement. At the time of recording, a wireless potentiostat was plugged-in to acquire oscillatory signals. The EEG and EMG data can be utilized to assess alterations in neuronal frequency and sleep staging. Critically, the EEG/EMG system exhibits 9-to-12-month longevity and does not impede mice movement, allowing simultaneous electrophysiological and behavioral testing. In the present study, we demonstrate a combination of EEG/EMG recordings with home-cage locomotion, sleep assessments at baseline and after disruption, nesting, and, notably, during cognitive testing in the Barnes maze. We propose this system is feasible for concurrent recordings in a multitude of behavioral tasks beyond those investigated in the current study. Created with BioRender.com.

Electroencephalography and behavioral testing have been frequently employed to research many disorders pre-clinically (Hamm et al., 2015; Pinnell et al., 2015, 2016; Xu et al., 2015; Kent et al., 2018; Medlej et al., 2019; Vorobyov et al., 2019; Buenrostro-Jáuregui et al., 2020; Garcia-Cortadella et al., 2021). Yet, in the study of aging and neurodegenerative disease, improving these techniques to preserve signal consistency over long periods of time is desirable. Furthermore, there is a need for these EEG recordings to be conducted in freely moving animals, facilitating a multitude of small and large arena behavioral tasks (i.e., Barnes maze) and providing a strong correlate of clinical assessments (Helfrich and Knight, 2019; Beppi et al., 2021). Herein, we employ a wireless EEG/EMG electrode headcap system to obtain high-quality longitudinal (up to 12 months post-surgery) EEG data from mice and describe the utility of combinatorial recordings during sleep, behavioral, and cognitive paradigms (Figure 1).

## 2. Materials and equipment

### 2.1. EEG headcap surgery

1. 2EEG/1EMG (stainless steel leads) headcaps (8201, Pinnacle Technology Inc.) – x1/mouse.
2. Anterior stainless steel electrode screws 0.10′′ (Pinnacle Technology Inc.) – x2/mouse.
3. Posterior stainless steel electrode screws 0.12′′ (Pinnacle Technology Inc.) – x2/mouse.
4. Flathead screwdriver, 1-mm head-size (available from Pinnacle Technology Inc.).
5. Stereotaxic frame, with nose cone for isoflurane and blunted ear bars.
6. Surgical tools (autoclaved or hot bead sterilized): scalpel handle and type #11 blade, fine forceps x3, curved forceps, iris scissors, syringes (1 and 3 mL), and needles (23 and 30 g), hemostat, cotton swab x3–5/mouse.
7. Adhesives: cyanoacrylate Vetbond (various suppliers), 2-part epoxy (Pinnacle Technology Inc.), dental acrylic powder and jet liquid OR other dental cement (various suppliers); weigh boats and paintbrushes.
8. Animal care: Systane Gel lubricant eye gel (Alcon), fur clippers, ethanol (70%), Betadine scrub, Betadine solution, heat pad for mice, Dermachlor Rinse (Butler), sutures, recovery cage, alcohol wipes, and weighing scale; anesthetic: isoflurane; analgesic: Metacam, 5 mg/kg [dilute 1:10; the quantity injected is mouse weight (g) divided by 100]; local anesthetic: Bupivacaine, 1–2 mg/kg 0.125% (dilute 1:2; 0.1 mL injections along incision site); sterile gauze x3/mouse and sterile saline.

### 2.2. EEG recording equipment

1. Reusable potentiostat, Bluetooth USB dongle, battery caps (8274, Pinnacle Technology Inc.). We have tested up to 4 simultaneous 8274 recordings, with a potential for upward of 16 on one computer depending on Bluetooth traffic.
2. Zinc air batteries (Size 13, PR48, 1.45V; available from Pinnacle Technology Inc.).
3. Sirenia^®^ Acquisition software (Pinnacle Technology Inc.).
4. Sirenia^®^ Sleep Pro (Pinnacle Technology Inc.).

### 2.3. Behavioral equipment

1. PhenoTyper home cage and computer (Noldus).
2. Barnes maze (Maze Engineers): spatial cues, escape box, overhead light (LED; 5000K, 4000 lumen), camera, and computer (Noldus).
3. EthoVision XT15 software (Noldus).

## 3. Methods

### 3.1. Animals

All animal experiments were conducted in accordance with the ethical standards of the Canadian Council on Animal Care guidelines and approved by the Animal Care Committee of CAMH (Protocol #850). Mice were housed in a 12-h light:dark-cycle with *ad libitum* access to chow and water. For longevity analysis, 12 C57bl/6J mice (sex-balanced) underwent headcap surgery at 3.5 months of age. Of the 12, 6 were transgenic *App^NL–F/NL–F^* knock-in mice bred in-house (Saito et al., 2014) and 6 were non-transgenic mice (Jackson Laboratory). Ordered mice were allowed to habituate to the facility for 1.5 months prior to data collection. Four additional C57bl/6J female mice bred in-house were utilized for the optimization and validation of the EEG/EMG surgical technique and data analysis. To compare dark-cycle mobility without headcap and potentiostat, one additional non-transgenic mouse was utilized (female; bred in-house). Surgical implantation was successful in transgenic and non-transgenic, for both female and male mice; no sex differences were reported for frequency distribution or longevity of signal (refer to results section 4.1). Table 1 summarizes mouse groupings utilized herein and the current surgical attrition rate (4.76%) for experiments following technical optimization and the completion of the present study. We report results with sex and genotype grouped together as our objective was to assess the longevity and viability of combining headcap recordings with behavior.

**TABLE 1:** Mouse groups utilized for optimization and analysis, and current attrition rates.

Mouse grouping	*N*	Sex	Analyses/Figures	Implant/Surgical concerns	Health/Aging concerns
**Longevity** (EEG/EMG headcap)	12 (50% *App*^NL–F/NL–F^) transgenic, 50% non-transgenic).	6M 6F	8/12 mice: Figures 3A–D and sex differences analysis (4M and 4F); 7/12 mice: Figures 3E–H; Other four mice used for further testing (see below).	1 headcap dislodged @ 5-months post-surgery due to worn-away cement (see section “4.5 Pitfalls, limitations, troubleshooting, and alternatives”).	1 malocclusion; 2 found dead; 1 sacrificed because skin grew under headcap.
**Testing + young mice (4**–**5 months)** (EEG/EMG headcap)	4 (extra from longevity; all non-transgenic)	2M 2F	4 mice: Figures 4, 6, 7; 3 mice: Figure 5; Supplementary data	None	See above; malocclusion endpoint before Barnes maze.
**Testing + aged mice (9**–**10 months)** (EEG/EMG headcap)	4	4F	4 mice: Figures 4–6; 2 mice: Figure 7 Supplementary data	Improper EMG placement on 2 mice (EMG data omitted; see section “3.2 EEG headcap surgical procedure,” step 19 for tips); 1 headcap dislodged @ 8-months post-surgery (after data collection) due to worn-away cement (see section “4.5 Pitfalls, limitations, troubleshooting, and alternatives”); 2 omitted from sleep disruption analysis due to battery issue.	None
**Assessing mobility** (no headcap)	1	1F	Supplementary data only	N/A	N/A
**Other mice used in optimization of the current study**	6	3M 3F	N/A	3 died in surgery; 1 sacrificed post-surgery (poor recovery).	N/A
**Current attrition rates** (for additional surgeries since completion of the current study)	**Total:** 3/42 mice (7.14%). **Surgery-related:** 2/42 mice (4.76%).	1M 2F	N/A	Surgery related endpoint. 1 died in surgery. 1 poor recovery.	Non-Surgery related endpoint. 1 weight loss.

The first five groupings document mice utilized in the present study to optimize this technique and assess its applications. The final row documents current attrition rates for experiments and surgeries conducted since the completion of this study (independent of the previous five groupings; no data shown from these mice), to provide an aid for experimental design.

### 3.2. EEG headcap surgical procedure

**Objective:** 2EEG/1EMG headcap implantation allowing longitudinal recordings. **Note:** The surgical procedure was adapted from the information made available by Pinnacle Technologies Inc.

1. Ensure surgical equipment is sterilized and proper aseptic technique is followed.
2. Anesthetize the mouse in an isoflurane chamber (5% induction, 1% oxygen) and then transfer them to nosecone delivery.
3. Lower isoflurane to 3% and monitor the depth of anesthesia (i.e., toe pinch and breathing); isoflurane can now be adjusted downward in 0.25% intervals while maintaining the anesthetic plane. Once on the stereotaxic frame, maintain isoflurane at 1.25–2% while monitoring for the depth of anesthesia. Alternate anesthetics may be utilized but were not tested herein.
4. Weigh the mouse and then inject analgesic Metacam, 5 mg/kg (diluted 1:10), and saline (0.5 mL) subcutaneously.
5. Apply Systane Gel ointment on the eyes.
6. Inject local anesthetic Marcaine (bupivacaine), 1–2 mg/kg 0.125% (diluted 1:2); subcutaneously at the incision site (∼0.1–0.2 mL).
7. Using the clippers, shave away the fur on the top of the head.
8. Align the mouse in the stereotaxic frame, ensure the skull top is flat, and use a heat pad to maintain body temperature.
9. Clean the incision site with betadine scrub, then 70% ethanol, and then betadine solution.
10. Make a ∼1.5-cm incision using the scalpel blade along the skull midline, starting posterior to eyes until just past lambda.
11. Using a cotton swab, pull the skin to the sides to reveal the skull surface to avoid curling.
12. Dip another cotton swab in 70% ethanol to dry the skull surface (this ensures a strong adhesion of Vet Bond with potentiostat).
13. **CRITICAL STEP:** To align the placement of the headcap, use forceps to position the headcap with anterior screws to be embedded in the skull on top of the prefrontal cortex [(Bregma −2–2.5 mm AP, 1.5 ML (over both hemispheres)] and posterior screws to be anterior to lambda [(Bregma 3.5–4 mm AP, 1.5 ML (over both hemispheres)] above the retrosplenial and visual cortices (see Figure 1). A dye can be used to help landmark screw placement. **Note:** when the mice are small (20–25 g) it is advantageous to place the headcap as anterior as possible (before the skull becomes too narrow) to ensure that both anterior and posterior screws are above the cortex. If the posterior screws are further back where the cortex falls away and/or above the cerebellum, EEG activity on these channels will be too low in amplitude. **Note:** right hemisphere anterior and posterior screws confer signal for EEG2 and EEG1 channels, respectively; left hemisphere anterior and posterior screws confer signal for ground and EEG common (for reducing noise in the EEG1 and 2 channels), respectively.
14. Apply 1–2 drops of Vet Bond to the underside of the headcap once positioning is landmarked. Try to use as little as possible in order not to cover the screw holes, and leave it for 10 min for drying/curing.
15. ** CRITICAL STEP:** Tap electrode screw holes with a 23 g needle, gently pushing down through the headcap holes to partially penetrate the skull surface. Rotate the needle lightly until there is mild resistance to create a pilot hole for the screw. Insert one screw at a time into the hole with fine forceps and use a 1-mm screwdriver to lower it into the hole. If it grips the skull (should feel minor resistance), then lower it until the screwhead is approximately halfway to the base of the headcap. If the screw is not gripping, continue to tap the hole with a needle and re-attempt the placement of the screw. Repeat for each screw until all 4 (2 anterior and 2 posterior) are in place.
16. Prepare epoxy fresh each time: mix 2 parts in a weigh boat with a paintbrush, stirring vigorously for 1 min. **Note:** Ensure epoxy is ready, this may require mixing or agitation of syringes first prior to application.
17. **CRITICAL STEP:** Apply a very small amount of epoxy to the screws using a fine gauge needle (e.g., 30 g needle on a 3-mL syringe as an applicator) on the outermost corner of the screw hole on the headcap so that it touches the threaded shank of the screw and connect the epoxy on the base of the headcap to the screwhead, ensuring a continuous seal. Repeat for each screw. Allow the epoxy to set for 15 min before applying dental acrylic. **Note:** do not let the epoxy from one screw contact another; a very minimal amount of epoxy is applied to not impede the signal.
18. Tighten screws so that the screwhead rests on the board base.
19. Placement of EMG wires in nuchal muscles: bend wires gently with fine forceps approximately half to two-thirds up so that the tip half is angled down (in line with how the neck is positioned in the stereotaxic frame) and laterally. With curved forceps make a pocket in the nuchal muscle and with fine forceps place the wire inside the pocket, ensuring that majority of the wire is within and covered by the muscle and not just under the skin. Repeat for the other wire.
20. **CRITICAL STEP:** Prepare dental acrylic/cement to apply to and insulate and protect screw electrodes: dip a small paintbrush in jet liquid and use a wet brush to pick up a small ball of acrylic powder, repeat dipping and picking up powder until the powder forms a gelatinous/viscous liquid ball for easier application. Apply to cover the screws and the base of the headcap, shaping it to continuously cover these parts and seal them to the skull.
  **(a) Note**: do NOT get acrylic on the top of the headcap (will block plugging-in of the potentiostat) or to the sides (will make it more difficult to grip the headcap when restraining the mice).
  **(b) Note**: covering the exposed edges of the EMG wires can help secure the wires and the headcap.
  **(c) Note**: over time (5+ months), the dental cement can wear away, so applying a full amount and ensuring the headcap is secured at the onset can help maintain the headcap placement. It is recommended to check every 3 months post-surgery and re-apply cement at least 1 week prior to collecting recordings.
  **(d) Note**: pull the skin away from cement to prevent it from fusing.
21. Suture the edges of the incision (typically only 1 suture is needed near the ears to close the skin above the back of the skull and the edges of EMG wires, check if an additional suture is necessary here and/or at the rostral end of the incision near the eyes).
22. Remove the mouse from the isoflurane and the stereotaxic frame and place them in a heated, clean cage for recovery.
23. During recovery: provide an additional injection of saline (0.5 mL) and Metacam analgesic [5 mg/kg (diluted 1:10)]. Use Dermachlor rinse or other topical antiseptics along the incision site and apply dropwise with minimal amounts.
24. Monitor recovery and post-op (additional analgesic as needed). Most mice recover within 30–45 min and exhibit normal behavior/are fully adapted to the headcap at 24 h. Wait 5–7 days before handling and recording.

### 3.3. EEG/EMG recording and alignment with behavioral assessments

**Objective:** To set up recordings in time with behavioral assays and ensure the quality of the signal.

1. **CRITICAL STEP:** Pre-handling of mice is highly recommended to habituate the mouse and reduce stress when the experimenter must restrain it to plug-in potentiostats. Place the mouse on a cloth and keep a light grip on the base of the tail. After a few seconds, grip the sides of the headcap with a straight hemostat while maintaining a grip on the base of the tail. Practice letting go of the tail and keeping the mouse restrained *via* the grip on the headcap. This is when the potentiostat would be plugged in. Repeat for 3–5 days, 1–2× per day. **Note:** if the mouse is aggressive and/or repetitively jerking their head, release until agitation has lowered and try again.
2. On the day of recording, prepare for recording: connect USB dongles to the computer and open Sirenia^®^ Acquisition software.
3. ** CRITICAL STEP:** Remove the cover from the zinc batteries and let it sit for 5 min. Put the batteries in a potentiostat and cover it with the battery cap positioned with holes allowing airflow to the zinc batteries. Battery life allows upward of 72-h of recording at 1,024 samples/s. It is advantageous to set up a potentiostat Bluetooth connection early in case there is battery failure; ∼1-h before plugging-in the mouse.
4. Restrain mice as described in step 1 and plug them in the potentiostat.
5. Connect the potentiostat to the Bluetooth USB dongle receiver in Sirenia^®^ Acquisition software. All data were sampled at 1,024 samples/s. EEG and EMG signals were acquired, digitized, and amplified at the potentiostat before being sent via Bluetooth to the USB receiver. Data were acquired at 100× gain and with 0.5 Hz (EEG1 and 2) and 10 Hz (EMG) high-pass filters, and a 500 Hz low-pass filter. EEG1 and 2 data were normalized to the EEG common electrode (left posterior screw), and the EMG signal was generated via the difference between the two wire leads.
6. ** CRITICAL STEP:** Check the amplitude range [50–250 μV is an ideal range for EEG and EMG channels, depending on wake vs. sleep: EEG has higher amplitude during sleep, especially NREM, and EMG has higher amplitude during wake and variable], especially on first recording post-surgery to determine any issues with the electrode and wire placement.
7. Sirenia^®^ Acquisition software encodes computer time within the file, allowing alignment with Noldus EthoVision XT software. EthoVision does not encode computer time but can be accurately controlled by the computer clock as set in the Trial Control Settings. For example, an experiment can be set to start accurately at a computer time of 12:00 in EthoVision, and by calculating the acquisition time, it can be linked to computer time as encoded by EEG/EMG data in Sirenia^®^.
8. Noldus PhenoTypers are home cages equipped with an overhead camera to monitor a variety of freely moving mouse behavior without the need for continued operator intervention. In the current application, this facilitated assessment of locomotion across a full circadian rhythm, collected in EthoVision XT. This provides a comprehensive sleep assessment in combination with EEG/EMG recordings. Other home-cage behaviors can be monitored alongside, including nesting as a measure of activities of daily living.

### 3.4. Barnes maze

**Objective:** To train mice to the location of the escape box (spatial learning) and assess their memory and neuronal activity during a probe recall trial (spatial memory). Seven mice were included in this analysis. Barnes maze was conducted similarly to what has been previously reported (Morrone et al., 2022; Xhima et al., 2022).

1. Around 1–2 weeks of mouse handling prior to Barnes maze assessment. For EEG/EMG mice, handle to familiarize the experimenter and mice, and utilize the technique described above to habituate the mice to plugging-in to the potentiostat.
2. A circular Barnes maze field (diameter: 92 cm; Maze Engineers; 20 holes) was utilized in a behavioral suite with an overhead camera connected to EthoVision XT15.
3. Testing Day 1: Mice were habituated to the Barnes maze and escape box, without spatial cues or the overhead light.
4. Overhead aversive light (brightness: 4,000 lumens; color range: 5,000 K) and spatial cues were included in all subsequent trials.
5. Testing Days 2–5: Mice were tested for learning in 2 trials per day (3-min trials with a 2-h inter-trial-interval). Once the mouse enters the escape box, the light was turned off and the trial ended. The latency to find the escape box (s) and the number of errors were calculated in EthoVision to assess learning.
6. Testing Day 6: Following a 2-day break, mice were tested for 5 min in a probe trial in which the escape box was blocked. Thirty minutes prior to the probe, the potentiostat was plugged-in to allow habituation before being placed in the Barnes maze. As described above for the PhenoTypers, Sirenia^®^ Acquisition and EthoVision software were run simultaneously. EEG/EMG data acquisition was started first (1 file per mouse) and later aligned to the clock start-time for the Barnes maze in the EthoVision raw data. The percentage of time spent searching in the correct quadrant was quantified in EthoVision.

### 3.5. Data analysis – Generating power and matching to the behavioral phenotype

Acquired EEG/EMG data was opened in Sirenia^®^ Sleep Pro and exported by mouse and the time segment to be analyzed. A 50–60 Hz digital filter (band-stop filter) was applied to sampled raw EEG and EMG signals to remove potential electrical interference in the generated power data as well as for sleep staging. Power (μV^2/Hz) data was then generated by filtering out low- and high-band frequencies to capture the following neuronal waves: alpha (8–13 Hz), beta (13–30 Hz), delta (0.5–4 Hz), theta (5.5–8.5 Hz), and gamma (35–44 Hz); EMG: 50–150 Hz. These are the default frequency bins in Sirenia^®^ Sleep Pro software which worked well for sleep staging in the present study, though there are options for adjusting these values (refer to section “4.5 Pitfalls, limitations, troubleshooting, and alternatives,” point #7 for further discussion). The full range was analyzed for each channel as well in 0.5–500 Hz (EEG1 and 2) and 10–500 Hz (EMG). Power was generated in 10-s intervals for assessing longevity and matching neuronal frequency to locomotion, and in 2-s intervals for matching neuronal frequency to performance in the Barnes maze. Power data for longevity analysis is presented as an average of power (generated in 10-s intervals) within a 30-min period or split by wake and sleep state then averaged.

Power data was exported as a .tsv file and imported into Excel for further analyses and synchronization with behavioral readouts. Specifically, this involved alignment of data by computer clock time embedded in both datasets allowing direct comparison of electrophysiological and behavioral parameters at set times. **Note:** this requires both Sirenia^®^ Acquisition and EthoVision XT to run simultaneously on the same computer.

The EEG2 neuronal power and EMG in 10-s epochs were averaged in 10-min bins to align to velocity (cm/s; velocity calculated in EthoVision as a 10-min average) for locomotor analyses; data were exported by mouse and the time segment to be analyzed: 7 p.m.–7 a.m. for locomotion dark cycle, 7 a.m.–7 p.m. for locomotion light-cycle.

For the Barnes maze, the entire recording was analyzed (5-min trial with ∼1 min of data preceding and following the test) and power was generated in 2-s intervals. EEG2 banded power was calculated as a percentage of full channel power and binned as target vs. non-target quadrants (based on location calculated in EthoVision), as well as 30-s baseline activity sampled from before and after the task. Data was further compared as power per band (alpha, beta, delta, theta, and EMG 50–150 Hz) between the target quadrant and non-target zones. Barnes maze power × location heat maps were generated in a representative mouse by first exporting raw positional data from EthoVision and averaging the 0.1-s *xy* data into 2-s intervals. The range of alpha and theta power was calculated to bin each 2-s interval as a proportion (0–10, 10–20, 20–30, 30–40, 40–50, 50–60, 60–70, 70–80, 80–90, and 90–100%) of total power. The location data was then graphed with the neuronal power indicated by size and color.

### 3.6. Generating representative data – EEG/EMG recordings to assess age-effects and response to PhenoTyper-mediated sleep disruption

**Objective:** To record EEG/EMG signal at baseline, during sleep disruption, and in a post-sleep disruption recovery period.

Young (4–5 months) and aged (9–10 months; refer to Table 1) mice underwent 24-h observation to capture a baseline circadian cycle of EEG/EMG recording. Mice were placed in PhenoTypers at 4 p.m. on the first day to allow habituation before recording onset at 7 p.m. (beginning of dark cycle). Video and EEG/EMG recordings occurred over 24 h (7 p.m. Day 1 – 7 p.m. Day 2), and mice were removed from the cage at 10 a.m. on the third day (after 42 h). **Note:** Both PhenoTyper and EEG systems allow recording up to 72 h, although only one full day was recorded herein. Nest images were captured at 18, 24, and 42 h and scored on a 1–5 scale (low-high complexity), as per Deacon (2006).

One week later, this paradigm was repeated with the addition of a 6-h sleep disruption (SD) period immediately before (12–6 p.m.). The top unit of PhenoTypers is additionally equipped with a white light and a speaker to elicit a tone (2,300 Hz, 80 dB). We used randomized white light and tone intervals as stimuli to reliably disrupt sleep, without direct handling of mice (Colavito et al., 2013). Specifically, every 30 s–3 min, the white light would turn on for a 20-s–1-min duration, and every 30 s–3 min, the tone would play for 10–30 s, for the 6-h SD period. These settings are applied in the Trial Control Settings in EthoVision, under Action: Top Unit (hardware control); refer to Supplementary Figure 2 for detailed settings. Notably, utilizing a home-cage environment with freely moving mice reduces stress, and PhenoTypers have built-in light and tone within each cage to increase reproducibility between animals and across studies.

**FIGURE 2:**
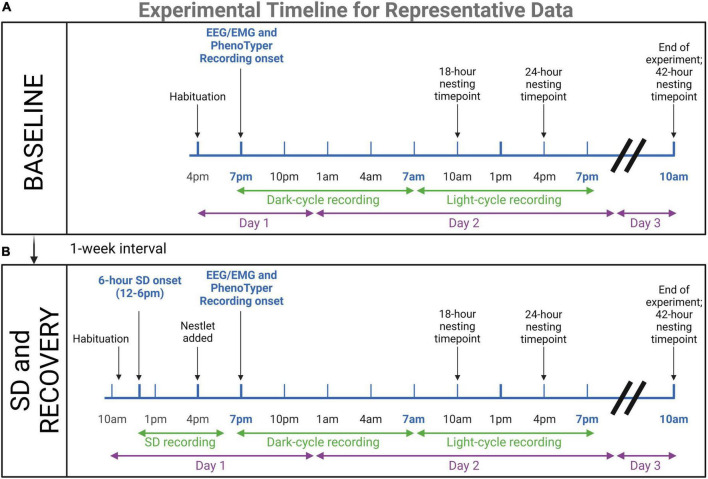
Timeline for representative data experiments. Young (*n* = 4) and aged (*n* = 4) mice underwent EEG/EMG recordings in PhenoTyper home cages to assess dark and light cycle sleep patterns and nesting. **(A)** 24-h baseline recordings were conducted, with 3 h of habituation prior to recording onset at 7 p.m. At 10 a.m. on the third day, mice were removed from the cage and potentiostats were unplugged. Nesting timepoints were 18, 24, and 42 h. **(B)** One week later, mice were tested again in the same paradigm with the addition of a 6-h sleep disruption (SD) prior to the recording. This SD was conducted in the home cages with aversive stimuli. Mice were plugged into potentiostats and habituated 1-h before the onset of SD (12–6 p.m.). The nestlet was added at 4 p.m. to keep nesting time points consistent. The immediate recovery period was recorded for 24-h to compare to baseline sleep patterns. The results of these experiments are presented as representative data of the utility of the concurrent electrophysiological and behavioral technique for age assessments (refer to Figures 6, 7). Created with BioRender.com.

Following SD, mice were immediately recorded by video and EEG/EMG for 24 h, and the nestlets scored, as in the baseline, facilitated a comparison of baseline and recovery after SD, as well as the effect of age. Nestlets were added part-way through the SD paradigm (4 p.m.). Figure 2 depicts the timeline of these experiments.

### 3.7. Data analysis: Sleep staging

Epochs were scored as REM sleep, NREM sleep, or wake in 10-s intervals by cluster scoring in Sirenia^®^ Sleep Pro; refer to Supplementary Figure 1 for representative stages and cluster scoring, similar to previously described methods (Kent et al., 2018). Theta/delta ratio was utilized to distinguish REM (high theta/delta ratio and low EMG) and NREM (low theta/delta ratio, high delta power, and low EMG) from wake (high and variable EMG). EEG2 theta power was divided by EEG2 delta power and graphed × EMG signal in the 50–150 Hz band to elucidate clusters of REM, NREM, and wake epochs. The remaining transitional epochs were scored manually. The accuracy of cluster scoring was validated per animal manually.

**For longevity:** 12-month post-surgical mice were scored in a 30-min period during the light cycle (∼3 p.m.) and utilized to bin power data by wake *vs*. sleep vigilance state.

**For representative data (baseline vs. recovery)**: full 12-h dark cycle (7 p.m. –7 a.m.) and light cycle (7 a.m.–7 p.m.) data were scored for REM, NREM, and wake stages from both the baseline and recovery after SD recordings. The proportion of epochs per stage was calculated for the full 12-h recordings, as well as in 2-h bins. Arousals [1–2 wake epochs (10–20 s) during sleep bouts] and micro-sleep shifts [1–2 sleep epochs (10–20 s) during wake bouts] were quantified during light-cycle baseline and recovery recordings and normalized to the sum of sleep and wake bouts, respectively. A bout was defined as 3+ continuous epochs, and the occurrence of 3+ continuous epochs of the opposite stage indicated the end of the current bout. NREM and REM were grouped for the calculation of sleep bouts.

**For SD:** the 6-h recording was scored, the proportion of epochs per stage calculated, and REM, NREM, and wake data were compared to the equivalent timespan during the baseline recording.

### 3.8. Statistical analysis

GraphPad Prism 9 was utilized for the generation of graphs and statistical analyses. Data are presented as mean ± SEM and best-fit line as ±95% CIs. Repeated measures of ANOVA and paired *t*-tests were utilized to assess longevity, Barnes maze analyses, nesting, sleep disruption, and movement with potentiostats. Unpaired *t*-test was utilized for comparing arousals and stage shifts across age. Where necessary, multiple comparisons were controlled for with Tukey (*F*-test statistics) and with Holm–Šídák (*T*-test statistics) *post hoc* tests. Linear and non-linear regression (exponential one-phase decay, least squares fit) were utilized to fit lines for neuronal frequency × locomotion. Two mice were graphed, but were omitted from the 12-month post-surgery statistical analysis due to the inability to distinguish sleep vs. wake; explained further in results text. In the neuronal frequency X locomotion dark-cycle data, 16 data points (out of 576 total) were excluded due to abnormally high EEG amplitudes in one mouse near the onset of recording; this did not persist. Two mice used in initial optimization were omitted from locomotion- and Barnes maze-associated EMG analyses, due to EMG signal degradation; the EEG2 signal reported was not affected. All values reported in the text (i.e., effect sizes) are mean ± SEM. Exact statistical tests and the number of individuals are reported in the results section and figure legends.

## 4. Results

We tested the application of *in vivo* wireless EEG/EMG recording during behavioral testing in mice as a proof-of-concept for assessing simultaneous electrophysiological and behavioral changes critical in aging and neurodegenerative research. Herein, we demonstrate the viability of recordings longitudinally, as well as during a variety of behavioral tasks and interventions, including home-cage locomotor assessment, activities of daily living, cognitive testing in the Barnes maze, and sleep staging at baseline and after automated sleep disruption (Figure 1).

### 4.1. Advantage #1: Quality signal can be ensured for longitudinal recordings up to 9 months post-surgery, with the potential for 12-month post-surgical sleep staging

**FIGURE 3:**
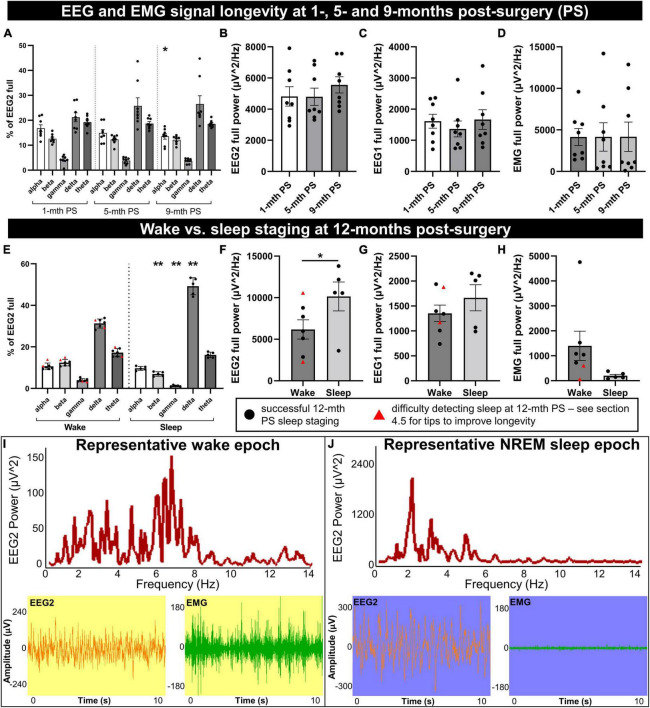
Longevity of EEG signal integrity until 9 to 12 months post-implantation. Mice were implanted with EEG/EMG headcaps, and recordings were collected at 1, 5, and 9 months post-surgery (PS) to look at signal longevity (*n* = 8), and sleep was staged at 12 months post-surgery (PS) (*n* = 7). Power was generated in alpha (8–13 Hz), beta (13–30 Hz), delta (0.5–4 Hz), theta (5.5–8.5 Hz), and gamma (35–44 Hz) bands for the anterior EEG electrode (EEG2), as well as full range (0.5–500 Hz) for EEG2, EEG1 (posterior) and EMG. **(A)** No overall effects of post-surgical time were detected on the proportion of neuronal frequency bands; differences between bands were significant on ANOVA. One significant *post hoc* comparison was detected between alpha banded power at 9 vs. 1-month post-surgery (*P* = 0.014). The full power of EEG2 **(B)**, EEG1 **(C)**, and EMG **(D)** channels did not significantly change between 1, 5, and 9 months post-surgery. At 12 months post-surgery, power was split by vigilance state for wake vs. sleep. **(E)** During sleep, there were proportionally fewer beta and gamma waves, and more delta than wake. Full EEG2 power was significantly increased **(F)**, EEG1 trended (*P* = 0.055) to increase **(G)**, and EMG trended (*P* = 0.097) to decrease **(H)**, in sleep compared to wake. Sleep was difficult to detect in two mice by cluster or manual staging (marked by red triangles), likely due to consistently low EMG. **(I,J)** Representative frequency distribution of wake and NREM sleep in 10 s epochs, with the corresponding EEG and EMG traces, demonstrate low amplitude/mixed frequency and high amplitude delta waves, respectively. Supplementary Figure 4 documents raw traces for REM, NREM, and wake and epoch frequency distribution for REM. Data are mean ± SEM. Two-way **(A)** and one-way **(B–D)** repeated measures ANOVA, multiple comparisons controlled for with Tukey *post hoc* test; multiple paired *t*-test with multiple comparisons controlled for with Holm–Šídák method **(E)**, paired *t*-test **(F–H)**. *P < 0.05, **P < 0.01.

Twelve 3.5-month-old mice were implanted with EEG/EMG headcaps to assess the potential for longitudinal recordings with the system. Locomotor home cage and EEG/EMG recordings were collected from 8 mice at 1, 5, and 9 months post-surgery, and 7 mice at the 12-month post-surgery at approximately 4-5, 8, 12, and 16 months of age, respectively (Figure 3). Four mice died from health-related issues (one was excluded only from the 12-month post-surgical analyses), and 1 had an issue with headcap stability after 8-month post-surgery testing, which could have been circumvented with re-application of cement. Table 1 annotates these details, and section “4.5 Pitfalls, limitations, troubleshooting, and alternatives” provides tips to improve stability/longevity. During recordings, mice adjusted well to the added weight of the potentiostat plugged in (∼3.4 g), exhibiting no differences in mobility when compared to mice without the potentiostat (refer to Supplementary Figure 3A).

The EEG power by frequency was generated on the anterior electrode for alpha (8–13 Hz), beta (13–30 Hz), gamma (35–44 Hz), delta (0.5–4 Hz), and theta (5.5–8.5 Hz) waveforms and normalized to the full channel signal during a predominant wake-period (Figure 3A). No overall significant differences were detected by 1, 5, and 9 months post-surgical time: *F* = 0.14, DFn, DFd = 1.55, 10.87, *P* = 0.82. Frequencies significantly differed from each other (*F* = 31.79, DFn, DFd = 1.15, 8.06, P = 0.0004), with no frequency × post-surgery interaction effect [*F* = 2.54, DFn, DFd = 1.80, 12.62, *P* = 0.12 (two-way repeated measures ANOVA)]. The *post hoc* analysis determined a significantly lower percentage of alpha power at 9 vs. 1 month post-surgery time (*P* = 0.014); no other comparisons differed (controlled for multiple comparisons with Tukey *post hoc* test; Figure 3A). No sex differences were detected in EEG frequency distribution at each post-surgery assessment (1 month: *F* = 0.045, DFn, DFd = 1, 6, *P* = 0.84; 5 months: *F* = 0.26, DFn, DFd = 1, 6, *P* = 0.63; 9 months: *F* = 0.023, DFn, DFd = 1, 6, *P* = 0.88; two-way repeated measures ANOVA; Figure 3A).

The full power for EEG2 (anterior electrode; *F* = 1.06, DFn, DFd = 1.28, 8.94, *P* = 0.35; Figure 3B), EEG1 (posterior electrode; *F* = 0.64, DFn, DFd = 1.31, 9.19, *P* = 0.49; Figure 3C), and EMG (*F* = 0.00038, DFn, DFd = 1.74, 12.17, *P* = 0.99; Figure 3D) were assessed (one-way repeated measures ANOVA), also distributing no significant differences at 1, 5, and 9-months post-surgery. No sex differences were detected for full power measures either (EEG2: *F* = 0.15, DFn, DFd = 1, 6, *P* = 0.71; EEG1: *F* = 0.28, DFn, DFd = 1, 6, *P* = 0.62; and EMG: *F* = 0.39, DFn, DFd = 1, 6, *P* = 0.56; two-way repeated measures ANOVA; Figures 3B–D). These data demonstrate the preservation of headcap signal integrity up to 9 months post-surgery. However, the loss of EMG signal is observed with increasing post-surgical time as the EMG leads can lose signal integrity due to the wear and tear of not being fixed in place, resting in the nuchal muscles. This does not necessarily preclude the differentiation of sleep and wake stages, which we demonstrate can be reliably measured at 12 months post-surgery in the same mice (Figures 3E–I).

Utilizing EEG and EMG signals, sleep vs. wake stages were identified in 10-s epochs for a 30-min period during the light-cycle, in 7 mice at 12 months post-surgery. Sleep was associated with significant reductions in the proportion of beta and gamma EEG2 signal (*t* = 7.43, df = 4, *P* = 0.0070 and *t* = 6.62, df = 4, *P* = 0.0081, respectively), increased delta (*t* = 9.58, df = 4, *P* = 0.0033), and no changes in alpha or theta (*t* = 0.92, df = 4, *P* = 0.65 and *t* = 0.84, df = 4, *P* = 0.65, respectively; Figure 3E) by multiple paired *t*-test (controlled for multiple comparisons with Holm–Šídák). Notably, increased delta frequency is characteristic of sleep (Brown et al., 2012). The full power of EEG2 significantly increased in sleep vs. wake (paired *t*-test: *t* = 4.30, df = 4, *P* = 0.013; Figure 3F), likely due to high amplitude delta waves, with a trend to increase in EEG1 full power (paired *t*-test: *t* = 2.68, df = 4, *P* = 0.055; Figure 3G). EMG full power trended to decrease in sleep vs. wake (paired *t*-test: *t* = 2.16, df = 4, *P* = 0.097; Figure 3H). Two of the mice, indicated by red triangles in Figures 3E–H, had no detectable sleep stages, which we concluded was due to the degradation of the EMG signal, and because the signal was consistently low (see Figure 3H), we were unable to differentiate sleep from wake and these mice were omitted from the paired statistical analyses in Figures 3E–H.

Using the actigraphy-like locomotion data collected in the PhenoTypers, we can differentiate sleep and wake stages by velocity (≤0. 1 cm/s for sleep) with 86.39 ± 3.35% (*n* = 2) accuracy to EMG, demonstrating the utility of acquiring locomotor and EEG data, as well as an alternative staging method to ensure the longevity of recordings.

Representative frequency distribution of wake and NREM sleep epochs at 12 months post-surgery demonstrate mixed frequency low-amplitude EEG signal and high-amplitude, low-frequency EEG signal, respectively (Figures 3I, J). REM sleep (high theta:delta ratio and low EMG) is also detectable at 12 months post-surgery (refer to Supplementary Figure 4A for representative EEG and EMG traces for all three stages and Supplementary Figure 4B for REM epoch frequency distribution).

When comparing neuronal power between animals, it may be best to plan for consistent post-surgery times and with normalization of power with another readout (i.e., as a percent of total EEG power, to locomotion or another behavioral readout). However, this data demonstrates that up to 9 months post-surgery, the quality of EEG and EMG signals was not significantly degraded and was sufficient for sleep staging, and can be successfully extended to 12 months post-surgery with proper troubleshooting (see section “4.5 Pitfalls, limitations, troubleshooting, and alternatives”).

For the EEG/EMG analyses in sections 4.2, 4.3, and 4.4, data from the four mice dropped from the longevity analyses was utilized along with 4 additional mice from our optimization and pilot studies (refer to Table 1). In light of no post-surgical or sex differences determined in signal longevity, female and male mice, and those with different post-surgical times were grouped together.

### 4.2. Advantage #2: Association of neuronal frequency with locomotor activity

**FIGURE 4:**
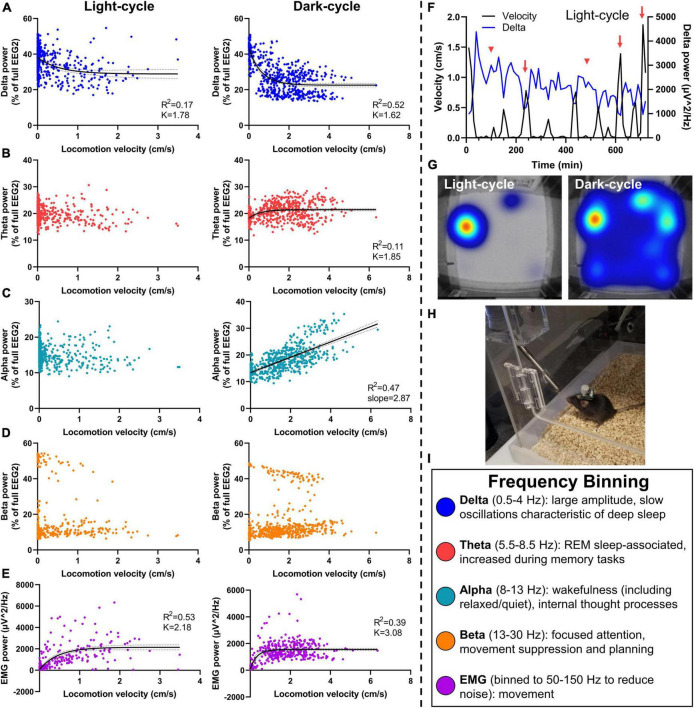
Relationship of neuronal frequency with locomotor activity. Mice were assessed in PhenoTypers with concurrent EEG/EMG recordings over 24 h in the light and dark cycles. Velocity was aligned with relative EEG frequency bands (normalized to full power) in 10-min bins to assess relationships between locomotion and sleep behavior with neuronal power. **(A)** Delta power was negatively associated with movement in light and dark cycles, demonstrating notably high delta during low-velocity recordings indicative of NREM and slow wave sleep, and a taper from delta power with high locomotion. **(B)** Theta power demonstrated a slight relationship in the dark cycle only. **(C)** A linear velocity × alpha relationship was detected in the dark-cycle only. **(D)** No velocity × beta relationships were observed. **(E)** EMG (50–150 Hz) exhibits a positive relationship with velocity in both light and dark cycles. **(F)** Light-cycle graph of velocity and delta power over time in a representative mouse demonstrates the increased delta power during sleep bouts (red arrowheads), and decreased delta power during movement (red arrows). **(G)** PhenoTyper heatmap of movement in the light and dark cycle. **(H)** Mice with EEG/EMG headcap and potentiostat exhibit normal exploratory and locomotor behavior in PhenoTyper (refer to Supplementary Figure 3A). **(I)** EEG frequency bins are listed with relationships to behavior. Data are 10-min bins of power and locomotor activity, with the line of best fit ±95% CIs, from *n* = 8 mice **(A–D)** or *n* = 6 mice **(E)**. Linear and non-linear regression (exponential one-phase decay, least squares fit).

We next binned neuronal power by frequency for delta (0.5–4 Hz), theta (5.5–8.5 Hz), alpha (8–13 Hz), and beta (13–30 Hz) waves, to assess for potential relationships with locomotion in PhenoTyper home-cage recordings in 8 mice; 2 mice were omitted from EMG analyses (refer to Table 1). Time-matched neuronal power (% of full power range) and velocity were graphed during sleep-dominant light-cycle and wake-dominant dark-cycle (Figure 4). Linear and non-linear regressions were conducted to fit power × locomotion relationships. An exponential one-phase relationship was observed with delta power and velocity during both light and dark cycles (df = 573, *R*^2^ = 0.17, Y0 = 38.02, plateau = 28.83, *K* = 1.78 and df = 557, *R*^2^ = 0.52, Y0 = 43.10, plateau = 22.56, *K* = 1.62, respectively; Figure 4A), demonstrating high delta power during periods of low velocity (likely sleep) and tapering off at higher velocities, especially during the dark-cycle. Delta waves are dominant during NREM and slow wave sleep (Figure 4I) (Brown et al., 2012).

No velocity × theta relationship was observed during the light cycle. We fit a one-phase line during the dark cycle (df = 557, *R*^2^ = 0.11, Y0 = 18.20, plateau = 21.44, *K* = 1.85; Figure 4B); a slight increase in the dark cycle perhaps demonstrated the relevance of theta waves in REM as well as wake-associated memory processes (Figure 4I) (Brown et al., 2012). Alpha power did not alter with velocity in the light cycle; yet, interestingly, exhibits a positive linear relationship during the dark cycle (*R*^2^ = 0.11, slope = 2.87, *Y*-intercept = 13.39, *F* = 501.4, DFn, DFd = 1, 558, *P* < 0.0001 for slope significantly non-zero; Figure 4C). This is likely a result of lower delta power and lesser sleep states in these higher velocity data points during the dark cycle, leading to a higher proportion of alpha, but not higher total alpha power. No linear relationship was observed between locomotion and total alpha power during the dark cycle (*R*^2^ = 0.00012, slope = −4.81, *Y*-intercept = 1151, *F* = 0.067, DFn, DFd = 1, 558, *P* = 0.80). Alpha waves are increased during wakefulness and thought processes and, therefore, indicative of greater activity. They can also associate with quiet wakefulness in which there is rest and reduced movement but not sleep (Figure 4I) (Brown et al., 2012), perhaps contributing to differing alpha × velocity correlations in the light- and dark cycles.

Beta power had no association during the light- or dark cycle (Figure 4D), indicative of the bimodal function of beta for movement inhibition, as would be observed with sleep onset, compared with attention and planning during wakefulness (Figure 4I) (Brown et al., 2012; Kropotov, 2016). Finally, we fit a one-phase decay line of EMG with velocity during the light and dark cycles (df = 429, *R*^2^ = 0.53, Y0 = 73.88, plateau = 2,149, *K* = 2.18 and df = 413, *R*^2^ = 0.39, Y0 = 82.20, plateau = 1554, *K* = 3.08, respectively; Figure 4E).

A representative time-based graph of velocity and delta power in the light cycle demonstrates increased delta during sleep bouts (low velocity) and vice versa (Figure 4F). When undisturbed, mice spend most of the light cycle in their nest (red–yellow hot-spot in the light cycle heat map), compared to high activity during the dark-cycle (broader heat map distribution in blue) (Figure 4G). PhenoTypers facilitate a controlled environment for consistent locomotor and EEG/EMG recordings, in which the mice are comfortable and have *ad libitum* access to food and water (Figure 4H). Preferably, these recordings are conducted in a dedicated testing room as the additional noise, vibrations, and electrical/Bluetooth interference from traffic in normal housing rooms can interfere with EEG/EMG signals.

### 4.3. Advantage #3: Association of neuronal activity with cognitive performance in the Barnes maze

**FIGURE 5:**
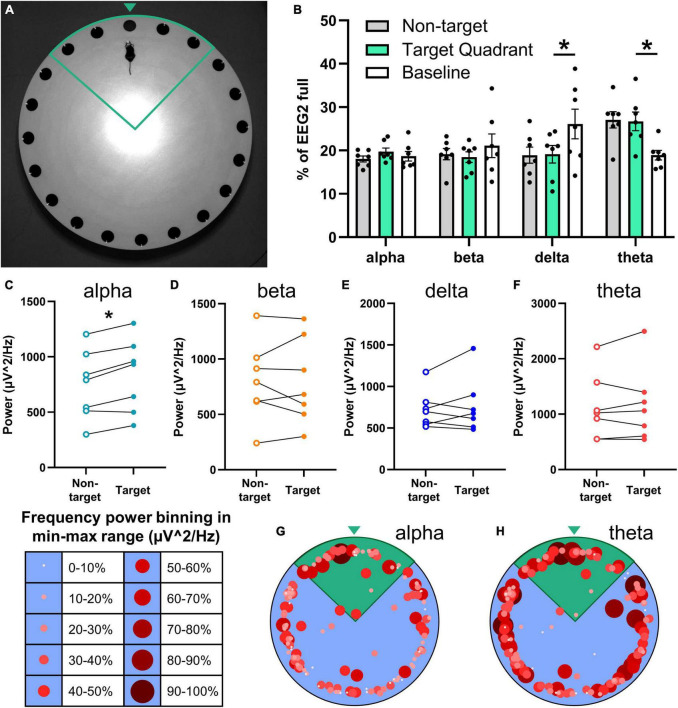
Relationship of the neuronal frequency with Barnes maze performance. EEG/EMG mice (*n* = 7) were tested in the Barnes maze as a proof-of-concept of combining recordings with cognitive testing. Mice learned the task successfully (refer to Supplementary Figures 5A,B) across four trial days (two trials per day) and were tested for spatial memory 2 days later in a probe trial in which the previously acquired escape location was blocked. **(A)** Mice with the potentiostat plugged-in navigate the Barnes maze successfully (refer to Supplementary Figure 3B), demonstrated here in the target quadrant (green outline) approaching the target zone (green arrowhead). **(B)** Neuronal power was generated in 2-s intervals, normalized to full EEG signal, and binned by mouse location in the Barnes maze (non-target and target quadrant), as well as at baseline (not performing task). While searching for the escape in the target quadrant, mice exhibit significantly lower delta power and significantly greater theta power than at baseline. Paired power comparisons of non-target vs. target quadrant determined significantly greater alpha power **(C)** in the target zone, and no differences for beta **(D)**, delta **(E)**, theta **(F)**, and EMG (refer to Supplementary Figure 5C) during the task. **(G,H)** Alpha and theta power were binned as a proportion (10% intervals) of the total power range recorded at each 2-s interval and graphed by size, color, and xy location in the Barnes maze. These heatmaps demonstrate higher alpha power in the target quadrant (green), and high theta throughout the maze. Representative heatmaps were generated in the same mice; positional data is the same for both. Data are mean ± SEM **(B)** or paired before-after comparisons **(C–F)**. Multiple paired *t*-tests with multiple comparisons were controlled for with the Holm–Šídák method. *P < 0.05.

To probe the viability of the wireless EEG/EMG system with additional behavioral assays, we assessed headcap mice in the Barnes maze, a test of spatial learning and memory (Figure 5). Mice successfully learned the location of the escape box based on spatial cues around the room (refer to Supplementary Figures 5A,B). Potentiostats were not plugged-in during learning trials in the present study because we anticipated the escape hole was not large enough (5 cm diameter) to accommodate the potentiostat. Utilizing larger escape holes or additional training time to help guide mice into the hole may facilitate EEG/EMG data collection during all trials. Three days following the last learning trial, potentiostats were plugged into mice headcaps, and mice were tested in a spatial memory probe in which the escape box was blocked. In this trial, mice were assessed for the percentage of time spent searching in the target quadrant during the 5-min trial (35.78 ± 4.10%; *n* = 7). Potentiostats did not affect mobility in the Barnes maze (refer to Supplementary Figure 3B). Representative image of a mouse with wireless EEG/EMG unit in the Barnes maze headed toward the escape location with target quadrant (green outline) and zone (green arrowhead) indicated in Figure 5A.

Neuronal power was analyzed by frequency in 2-s bins and aligned with the location in the Barnes maze in 7 mice. Frequency distribution was generated for neuronal power when mice were in the Barnes maze searching in non-target zones, in the target quadrant, and baseline activity before and after the task (Figure 5B). Target quadrant vs. baseline comparisons determined significantly less slow wave sleep-associated delta waves (*t* = 3.32, df = 6, *P* = 0.047) and greater theta power (*t* = 3.84, df = 6, *P* = 0.034) when mice were searching for the escape; alpha (*t* = 0.81, df = 6, *P* = 0.70) and beta (*t* = 0.76, df = 6, *P* = 0.70) waveforms did not change (multiple paired *t*-test; controlled for multiple comparisons with Holm–Šídák).

We assessed total power by frequency to further probe the potential relationship of neuronal power with performance in the Barnes maze. We observed a significant increase in alpha power in the target compared to non-target zones (*t* = 4.59, df = 6, *P* = 0.015; Figure 5C), but not in beta (*t* = 0.086, df = 6, *P* = 0.93; Figure 5D), delta (*t* = 0.80, df = 6, *P* = 0.84; Figure 5E) or theta (*t* = 0.50, df = 6, *P* = 0.87; Figure 5F) waves (multiple paired *t*-test; controlled for multiple comparisons with Holm–Šídák). EMG power (in 50–150 Hz) did not change in the target quadrant vs. non-target (*n* = 5; *t* = 1.36, df = 4, *P* = 0.25; paired *t*-test; refer to Supplementary Figure 5C).

In sum, higher alpha power was observed when mice were in the target quadrant and close to checking the correct hole, and higher theta power was observed throughout the task; these frequencies are associated with memory, thought, and attention (Figure 4I) (Brown et al., 2012; Kropotov, 2016). To visualize the association of neuronal frequency with the location in the Barnes maze, the power range of alpha and theta waves were separated into 10% bins and graphed by the *xy* location (Figures 5G, H). These representative images demonstrate higher selectivity of increased alpha neuronal power closer to the escape location, whereas theta power was less discriminate but high throughout the task. These data demonstrate the feasibility of EEG/EMG recordings simultaneous to behavioral testing in rodents. We anticipate that the combination of these techniques can help pinpoint electrophysiological alterations as they relate to cognitive decline and behavioral alterations across age and during the course of neurodegenerative disease progression.

### 4.4. Representative data: Sleep staging in young vs. aged mice at baseline and following sleep disruption

**FIGURE 6:**
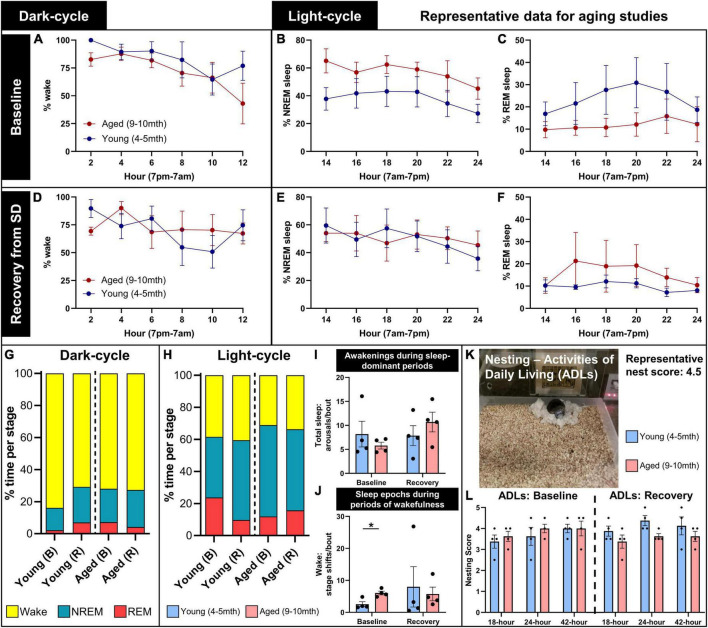
Representative data for aging studies on sleep and home-cage assessments. Young (4–5 months; *n* = 4) and aged (9–10 months; *n* = 4) mice were recorded for a full circadian cycle in PhenoTyper home cages with EEG/EMG recording from wireless headcaps. Recordings were conducted at a baseline, and in a recovery period immediately following 6-h sleep disruption (SD), 1 week later (refer to Figure 2 for timeline). Sleep was staged as wake, NREM, and REM in 10-s epochs from EEG/EMG recordings, split into 12-h dark and light cycles and analyzed in 2-h intervals. **(A)** During the baseline dark cycle, young and aged mice spend a majority of their time awake, and start to sleep more near the onset of the light cycle. **(B,C)** During the baseline light cycle, young mice trend to less NREM and more REM sleep than aged mice. **(D–F)** In the recording period immediately following SD (indicated here as recovery), all mice trend to more sleep during dark-cycle, and young mice trend to more NREM and less REM sleep during light cycle, compared to baseline. **(G,H)** Summary of the distribution of wake (yellow), NREM (blue), and REM (red) in young and aged mice in baseline (B) and recovery (R) recordings, for dark- and light-cycles. **(I)** During light-cycle, no significant differences in sleep arousals were detected between young and aged mice at baseline and recovery; **(J)** however, aged mice exhibited significantly more sleep epochs during periods of wakefulness at baseline (*P* = 0.022), indicating increased sleep fragmentation with age. **(K,L)** Nest building was scored at 18, 24, and 42 h as a measure of activities of daily living during baseline and in the post-SD recovery period, demonstrating the trend to reduced nest complexity in aged mice in the recovery period, compared with young mice (*P* = 0.081). Data are mean ± SEM **(A–F,I,J,L)** and mean **(G,H)**. Multiple unpaired *t*-tests with multiple comparisons controlled with the Holm–Šídák method **(I,J)**; two-way repeated measures ANOVA **(L)**. *P < 0.05.

We assessed young (4–5 months) and aged (9–10 months) mice with EEG/EMG headcaps to measure neuronal activity and locomotion in Noldus PhenoTypers across a full circadian cycle (24 h) at a baseline starting from the onset of their dark cycle (7 p.m.). One week later, the same mice were subjected to a 6-h sleep disruption (SD; 12–6 p.m.) *via* a loud tone and bright light inside each PhenoTyper cage, set on a procedurally randomized timer. Mice were measured for the next 24-h cycle after SD to assess the recovery period (refer to Figure 2 for timeline). Sleep was staged across the entire recording at baseline and in the recovery period as a representative of alterations across age and after SD (Figure 6).

At baseline during the dark cycle, young and aged mice spend the majority of time awake and start to sleep more as the onset of the light cycle approaches (7 a.m.; Figure 6A). During the baseline light cycle, mice sleep approximately 60% of the time, and young mice trend to more REM and less NREM sleep than aged mice (Figures 6B, C). Immediately following SD, mice were tested again to assess their sleep activity during the recovery period. More variability between sleep and wake states was observed during the recovery dark cycle, and young mice trended to sleep more compared with the baseline (Figure 6D). Slight decreases in sleep time were observed in the recovery period for young and aged mice, with less REM sleep in young mice (Figures 6E, F). Figures 6G, H summarizes these trends.

We analyzed for wake arousals detected during sleep-dominant periods in the light cycle at baseline and in the recovery period. Arousals did not significantly differ between young and aged mice at baseline (*t* = 0.86, df = 6, *P* = 0.60) or recovery (*t* = 0.97, df = 6, *P* = 0.60; multiple unpaired *t*-tests with multiple comparisons controlled for with Holm–Šídák method; Figure 6I), though it may increase in aged mice after SD (*t* = 1.84, df = 3, *P* = 0.16, paired *t*-test; effect size: 2.05× ± 0.59 increase). We then analyzed for micro-sleep episodes detected during wake-dominant periods in the light cycle, which were determined to be significantly more in aged mice at baseline (*t* = 3.63, df = 6, *P* = 0.022), but there were no differences in the recovery period (*t* = 0.34, df = 6, *P* = 0.75; Figure 6J). These data demonstrate increased sleep fragmentation with age.

Nesting was assessed during circadian recordings as a measure of activities of daily living. A representative image demonstrates a mouse with a headcap and potentiostat in the PhenoTyper home cage with a complex nest built (score: 4.5/5; Figure 6K). Nest complexity was scored at 18, 24, and 42 h during baseline and post-sleep disruption (recovery) recordings in young and aged mice. At baseline, young and aged mice trend to higher nest scores over time (*F* = 4.59, DFn, DFd = 1.27, 7.62, *P* = 0.060), with no differences between ages (*F* = 0.30, DFn, DFd = 1, 6, *P* = 0.60). During the recovery period, aged mice trend to lower nest complexity than young mice (*F* = 4.39, DFn, DFd = 1, 6, *P* = 0.081; two-way repeated measures ANOVA; Figure 6L). We anticipate that this is indicative of a loss of activities of daily living after lifestyle stressors, and how over-age these stressors and a diminished ability to compensate for them can potentially exacerbate behavioral deficits (Webb et al., 2018).

**FIGURE 7:**
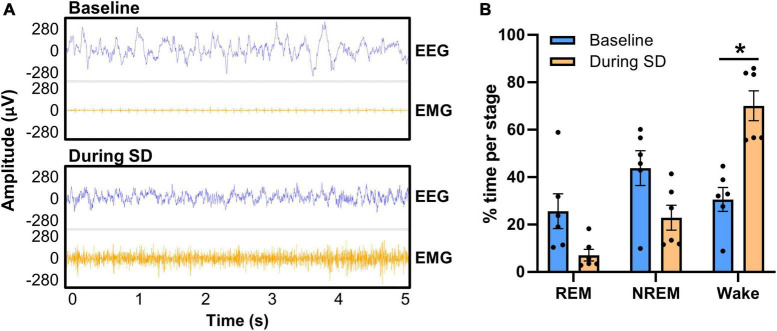
Confirmation of PhenoTyper-mediated sleep disruption in EEG. Mice (*n* = 6) underwent a baseline recording and an acute, 6-h sleep disruption (SD; 12–6 p.m.) 1 week later (refer to Figure 2 for timeline). SD involved an intermittent tone and light from the top-unit of the PhenoTyper so that each mouse was exposed to the same stimuli. The interval between both tone and light was procedurally randomized to prevent habituation. **(A)** Neuronal activity was recorded by EEG/EMG headcaps during SD and resembles wakefulness, with higher frequency EEG and EMG than sleep (baseline). Heartbeat can be detected in EMG when the mice are at rest [as in the baseline EMG in panel **(A)**], but is not always present. **(B)** EEG/EMG data was staged as NREM, REM, and wake in the same time frame at baseline and SD, demonstrating a reduction in the time spent asleep and a significant increase in wakefulness (*P* = 0.022). Data are mean ± SEM. Multiple paired *t*-tests with multiple comparisons were controlled for with the Holm–Šídák method. *P < 0.05.

We next confirmed in EEG that sleep was disrupted utilizing the tone and bright light in the PhenoTypers (Figure 7). Representative 5-s EEG and EMG traces demonstrate high amplitude slow waves during sleep (baseline) with tonic and low EMG amplitude, in contrast to the higher frequency, lower amplitude neuronal activity, and high EMG signal during SD, indicative of wakefulness (Figure 7A). Six out of the eight mice in Figure 6 were utilized for SD confirmation; the other 2 mice had battery issue causing data loss for half of the SD period, though they still exhibited disrupted sleep (refer to section “3.3 EEG/EMG recording and alignment with behavioral assessments,” step 3 for tips on ensuring battery quality). Assessment of REM, NREM, and wake staging during the 6-h SD, compared with time-matched baseline (multiple paired *t*-test with multiple comparisons controlled for with Holm–Šídák method), indicates significantly more wakefulness during the PhenoTyper-mediated SD (*t* = 4.34, df = 5, *p* = 0.022), with 55.29 ± 10.10% (*n* = 6) less time spent asleep (Figure 7B). Time in the REM (*t* = 2.10, df = 5, *p* = 0.17) and NREM (*t* = 1.79, df = 5, *p* = 0.17) stages trended to less during SD compared with baseline (Figure 7B).

These data indicate that randomized auditory and light stimuli in PhenoTyper cages are sufficient to disrupt sleep. We propose this method of sleep disruption recapitulates fragmenting of sleep that occurs with age (Scullin and Bliwise, 2015), instead of complete deprivation, and that an impaired ability to recover after sleep loss is a critical readout to understand how mounting sleep deficits may impact neurodegenerative diseases (Musiek and Holtzman, 2016).

### 4.5. Pitfalls, limitations, troubleshooting, and alternatives

1. The headcap procedure is feasible but is more challenging in female mice and mice under 3 months due to size. Consequently, the placement of the headcap needs greater precision to ensure that the anterior and posterior electrode screws are above the cortex.
2. We have observed 2 mice (out of 58 successful implantations; 3.45%) lose a headcap, at 5 and 9 months post-surgery. Both were identified as having reduced dental cement securing the headcap compared with mice that had surgeries at the same time. Dental cement can degrade over time, so it is critical to ensure coverage of screws and the base of the headcap at the time of surgery. A potential re-application of dental cement (every 3–6 months) applied under anesthesia may be necessary for longitudinal experiments in mice to prevent the headcap from getting displaced, or if EMG wires become exposed (re-cover connection to headcap to prevent further damage). This is quick (5–10 min/mouse), yet will depend on the time for the cement to set. Silicone coating of the exposed screwheads before dental cement re-application may also preserve signal longevity, although this was not tested herein.
3. For longitudinal experiments, it is important to consider attrition of animals *via* death from non-headcap-related causes, when considering sample size, in addition to the potential loss or displacement of the headcap.
4. To help preserve EEG signal quality over time (refer to Figure 3), an alternative method is to utilize screws with a wire attached (8403, Pinnacle Technology Inc.), allowing soldering of the electrode to the headcap.
5. If there are issues in restraining the mice by the headcap for plugging-in in the potentiostat (i.e., trouble gripping the headcap from dental cement, agitated mice), a fast-acting and clearing inhalational anesthetic can be used. The investigator can utilize isoflurane restraint (5%) for 1–2 min, just for induction. Mice recover quickly but should be given ∼1-h post-anesthesia prior to data collection.
6. Utilizing the cluster scoring method in Sirenia^®^ Sleep Pro leaves a few unscored transition epochs (refer to Supplementary Figure 1). Therefore, some manual scoring is necessary, but is quick.
7. The power data in the present study were generated utilizing the default frequency bins in Sirenia^®^ Sleep Pro, which were well-aligned to behavioral and vigilance states, and advantageous for separating NREM and REM sleep epochs. However, an overlap exists between 8 and 8.5 Hz in our theta and alpha frequency bins, and there is potential for biologically relevant neuronal activity between 4 and 5.5 Hz being missed in the present analyses. Frequency binning can be edited in the software, allowing additional bands such as sigma [prominent in sleep spindles; 12–16 Hz (Holz et al., 2012)] and gamma (included by default; 35–44 Hz); albeit, the power of this low gamma range was low in our longevity analysis during wake and sleep states (refer to Figures 3A, E), and was hence omitted for subsequent analyses. Sirenia^®^ Sleep Pro also allows manually defined sleep staging, which, for example, can allow the identification of slow wave sleep (by breaking down NREM by delta power) and quiet wakefulness (low EMG, low delta, and high alpha). This can be accomplished with manual, cluster, or threshold scoring, which allows users to define rules for scoring based on neuronal frequency power and EMG.
8. Alternatively, Freyburger et al. (2016) utilized shorter epochs (i.e., 4 s compared to 10 s in the present study) and analyzed power spectra by vigilance/sleep stages (REM, NREM, and wake) (McShane et al., 2010), which is advantageous in detecting short arousals and neuronal frequency changes by stage, respectively.
9. The exported data can be utilized to align other electrophysiological readouts to the behavioral phenotype, including coherence between the two EEG channels, phase synchronization, and cross-frequency coupling (Tort et al., 2009; Canolty and Knight, 2010; Hata et al., 2016). An alternative is to export EEG/EMG data acquired in Sirenia^®^ as a .edf file for analysis in other software.
10. Alternate headcap systems: (1) Pinnacle offers the option for depth electrodes, advantageous in adding regional field potential recordings (i.e., from the hippocampus). (2) Spike Gadgets offers headcaps with a 32-channel capacity. (3) For other EEG systems, refer to Pinnell et al. (2015, 2016), Zayachkivsky et al. (2015), Vogler et al. (2017), Kent et al. (2018), Crouch et al. (2019), Medlej et al. (2019), Buenrostro-Jáuregui et al. (2020). A recent study conducted by Crouch et al. (2019) demonstrated the utility of combined EEG and behavioral assessments in phenotyping mouse models of AD. This technique provides greater temporal resolution than what we demonstrate herein; however, the possibility for longitudinal-based recordings as we describe is advantageous.
11. Chronic PhenoTyper-mediated sleep disruption was not tested with EEG/EMG recording. It is possible that mice adjust to the randomized tone and light stressors after continued exposure, in which case shorter intervals and longer tone/light lengths could reduce habituation, or alternatively, the usage of different sleep disruption techniques might be preferable (i.e., rotating bar and gentle handling) (Colavito et al., 2013).
12. The synchronization of behavioral and electrophysiological data herein was conducted utilizing the computer clock, with both software running on the same computer, and was accurate to the second. Higher precision can be attained with alternative methods: transistor–transistor logic (TTL) signals for data alignment are possible with both systems.
13. Though we observe no significant technical differences up to 9 months post-surgery, it is recommended to consider consistent post-surgical time in the study design.

## 5. Discussion

Herein, we extensively describe and vet a method for concurrent wireless EEG/EMG recording with untethered, freely moving testing, including alignment of neuronal oscillatory signals to the behavioral phenotype. A one-time headcap surgery provides sustained signal longevity of at least 9 months, with a high potential for 12-month post-surgery that would be useful for experimental modeling of sleep staging, especially for facilitating longitudinal experimentation, critical in aging and neurodegenerative disease research. We found that the headcap and the wireless potentiostat recording unit did not impede mouse locomotion, allowing synchronization with a variety of behavioral assays, which we demonstrate for locomotion and home-cage PhenoTyper assessment, as well as cognitive performance in the Barnes maze.

We anticipate this procedure could be used in a majority of rodent behavioral tasks without the need to adapt the task to accommodate a tethered system, particularly advantageous in large arena assays (Barnes maze, open field), and without the concern of wire entanglement in walled assays (elevated plus maze, shelter/cognitive choice hole entries in PhenoTypers). However, some minor physical constraints exist with the wireless system, such as the size of the potentiostat impeding entry into the hole. This can be accommodated by using larger entry holes or longer training periods. Caution should also be considered in water-based assays, such as the Morris water maze as the device may not be waterproof (Pinnell et al., 2016).

For home-cage assessments, this system is particularly advantageous in that a full circadian rhythm could be captured in a single recording (with upward of 72 h before changing batteries), allowing the possibility for the recording of multiple days of sleep staging in one subject without intervention. This, paired with the consistent PhenoTyper-mediated sleep disruption paradigm we describe, can help elucidate sleep resilience in response to stress and deprivation, as well as how this is altered with disease and age (Brieva et al., 2021; Cai et al., 2021; Casale et al., 2021).

We identified suggestive relationships between neuronal frequency bins with locomotion and cognitive performance, which warrant further investigation. Delta power was associated with sleep bouts as evidenced by negative non-linear relationships with locomotion in light and dark cycles, and the proportion of alpha power was more dominant compared to other frequencies at higher locomotion states in the dark cycle, likely due to the reduced power of sleep-associated delta waves (Brown et al., 2012). While conducting the Barnes maze cognitive task, mice exhibited decreased delta and increased theta power compared to the baseline activity, indicative of a higher activity/vigilance state (Brown et al., 2012), and of memory and navigation (Buzsáki and Draguhn, 2004; Buzsáki and Moser, 2013; Jung and Carlén, 2021), respectively. Hippocampal and entorhinal cortical theta is typically associated with spatial memory performance, though the contribution of widespread cortical theta oscillations, including the prefrontal cortex (under our anterior electrodes), contribute to this cognitive processing (Buzsáki and Draguhn, 2004; Buzsáki and Moser, 2013; Jung and Carlén, 2021). Our results also suggest higher alpha power occurs near the target zone in the Barnes maze, which may relate to internally directed thought processes (Brown et al., 2012), as the mice search more precisely near where it anticipates the escape box. The contributions of EEG and neuronal waveforms to behavior have been previously reviewed (Brown et al., 2012; Kropotov, 2016; Helfrich and Knight, 2019; Beppi et al., 2021), whereas our results indicate the utility of acquiring oscillatory signals during behavioral testing in rodents. Notably, this technique facilitates the identification of electrophysiological alterations as they directly relate to the cognitive/behavioral phenotype in aging and neurodegenerative preclinical models, which may elucidate subtle or early markers of disease. The robustness of these observations could be important for future research, especially for predicting disease onset or progression.

The use of EEG as a physiological biomarker for clinical diagnosis and prognosis has become increasingly popular in recent years (Keizer, 2021). Significant differences in EEG activity have been described in neurodegenerative conditions such as AD, Parkinson’s, and frontotemporal lobe dementia (Babiloni et al., 2011, 2020; Garn et al., 2015; Goossens et al., 2017). Specific EEG markers have been shown to correlate with AD severity and provide differential dementia diagnosis (Garn et al., 2015; Goossens et al., 2017). The value of EEG biomarkers extends to mood disorders such as depression (Kaiser et al., 2018; Dev et al., 2022), anxiety disorders (Pavlenko et al., 2009; Al-Ezzi et al., 2021), and neurodevelopmental disorders such as Attention-Deficit/Hyperactivity Disorder and Autism Spectrum Disorder (Wang et al., 2013; Matlis et al., 2015; Angelidis et al., 2016). The use of EEG in brain disorders may complement traditional diagnostic methods of structured interviews and questionnaires (Snyder et al., 2015; Keizer, 2021), and the ability to detect and characterize these biomarkers may prove essential for preclinical research.

Finally, EEG is frequently employed as a tool to understand human cognition (Helfrich and Knight, 2019; Beppi et al., 2021), indicating the importance of preclinical correlates to understand cognitive and electrophysiological impairments in both healthy aging and disordered brain states. We propose that the full cognitive assessments and freely moving electrophysiological data documented herein will improve the validity of research into a majority of brain disorders. A description of limitations and alternative approaches is included in section “4.5 Pitfalls, limitations, troubleshooting, and alternatives.”

We anticipate the method described herein for concurrent EEG and behavioral testing in mice will be highly advantageous in preclinical research in a multitude of aging, neurological, and psychiatric disorders, including Alzheimer’s and Parkinson’s diseases, frontotemporal dementia, depression, and schizophrenia, all of which involve neuronal dysfunction and sleep dysregulation (Liu et al., 2004; Ju et al., 2014; Chahine et al., 2017; Fang et al., 2019; Waite et al., 2020; Wainberg et al., 2021), and will benefit from the alignment of neuronal oscillatory-to-behavioral deficits. Finally, the advent of investigating longitudinal readouts can help in the identification of predictive EEG biomarkers of cognitive decline and the pathophysiology of diseases like Alzheimer’s.

## Data availability statement

The raw data supporting the conclusions of this article will be made available by the authors, without undue reservation.

## Ethics statement

All animal experiments were conducted in accordance with the ethical standards of the Canadian Council on Animal Care guidelines and approved by the Animal Care Committee of CAMH (Protocol #850).

## Author contributions

CM and WY designed the study. CM and AT collected the data. CM, AT, and SG conducted data analysis. CM, EC, and WY wrote the manuscript. All authors edited and revised the manuscript, contributed to the article, and approved the submitted version.

## Glossary

Barnes maze: a cognitive task for spatial learning and memory in mice; Bout: a period of time spent asleep or awake, defined herein as three continuous 10-second epochs; EDF: European Data Format; a standard time series file format commonly used for wave-form data such as EEG and EMG; Epoch: period of time of wave-form data utilized to stage sleep (10-seconds herein); EthoVision XT: Noldus behavior acquisition and analysis software; Non-rapid eye movement (NREM) sleep: sleep stages including restorative slow wave sleep, characterized by delta waves in EEG; PhenoTyper: a home-cage designed for continuous video assessment of mice; Potentiostat: recording device connected to the headcaps to wirelessly acquire and transmit EEG and EMG data; Rapid eye movement (REM) sleep: sleep stage involved in memory consolidation and dreaming, distinguished from NREM by increased theta and reduced delta waves in EEG; Sirenia^®^: Pinnacle acquisition and analysis software for EEG and EMG data; Transistor-transistor logic (TTL): for circuit integration, can be used to align electrical signal within multiple software; tsv: tab-separated values text file.
