# Supplementary material for: Concurrent behavioral and electrophysiological longitudinal recordings for in vivo assessment of aging
Source: Front Aging Neurosci. 2023 Jan 18;14:952101. doi: 10.3389/fnagi.2022.952101 (PMC9891465; doi:10.3389/fnagi.2022.952101)
Supplement: Supplementary file 1 [file Data_Sheet_1.PDF]

Concurrent behavioral and electrophysiological longitudinal recordings for *in vivo* assessment of aging

Supplementary Material

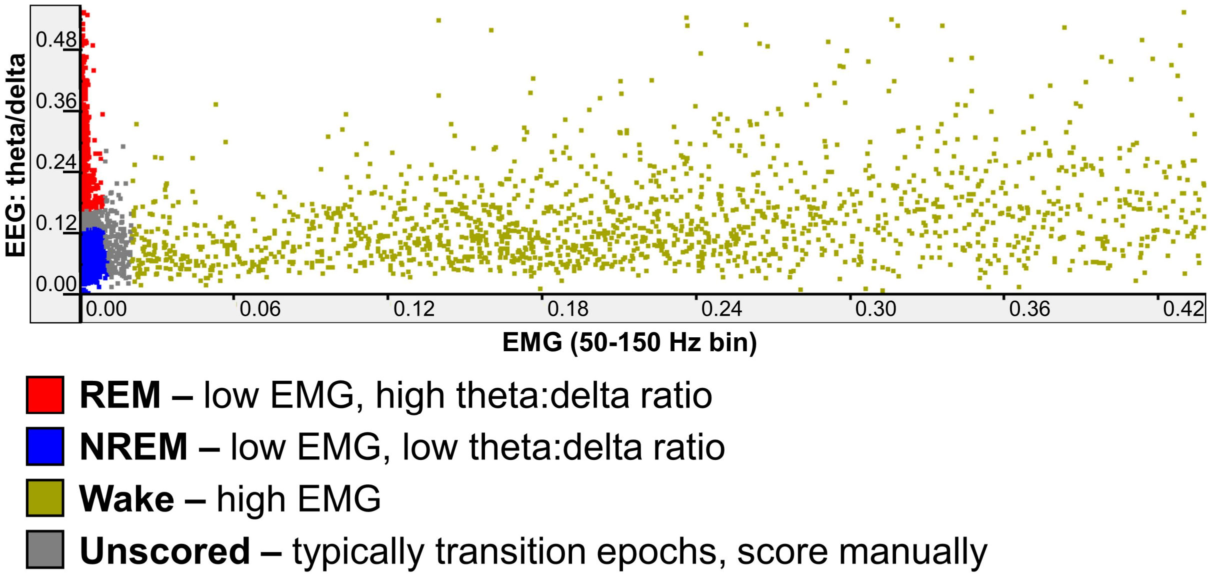

Supplementary Figure 1: Sleep staging in Sirenia® Sleep Pro. REM, NREM and wake are staged by epoch. Epoch length utilized in the present study was 10-seconds. Representative image of cluster sleep scoring method in Sirenia® Sleep Pro for a 12-hour light-cycle recording. After generating power data, EMG (50-150 Hz) was graphed on the x axis and theta/delta (from EEG2) on the y axis. This allows quick selection of REM (red; x<0.01, y>0.16) NREM (blue; x<0.01, y<0.12) and wake (yellow; x>0.02). There are a few unscored epochs (gray) which can be scored manually. These are typically transition epochs in which the mouse undergoes a stage transition within the 10-second epoch. Values >~0.42 on x axis and >~0.48 on y axis were cropped out of this image, but included in analysis as wake and REM, respectively. Image captured after analysis performed on Sirenia® Sleep Pro software.

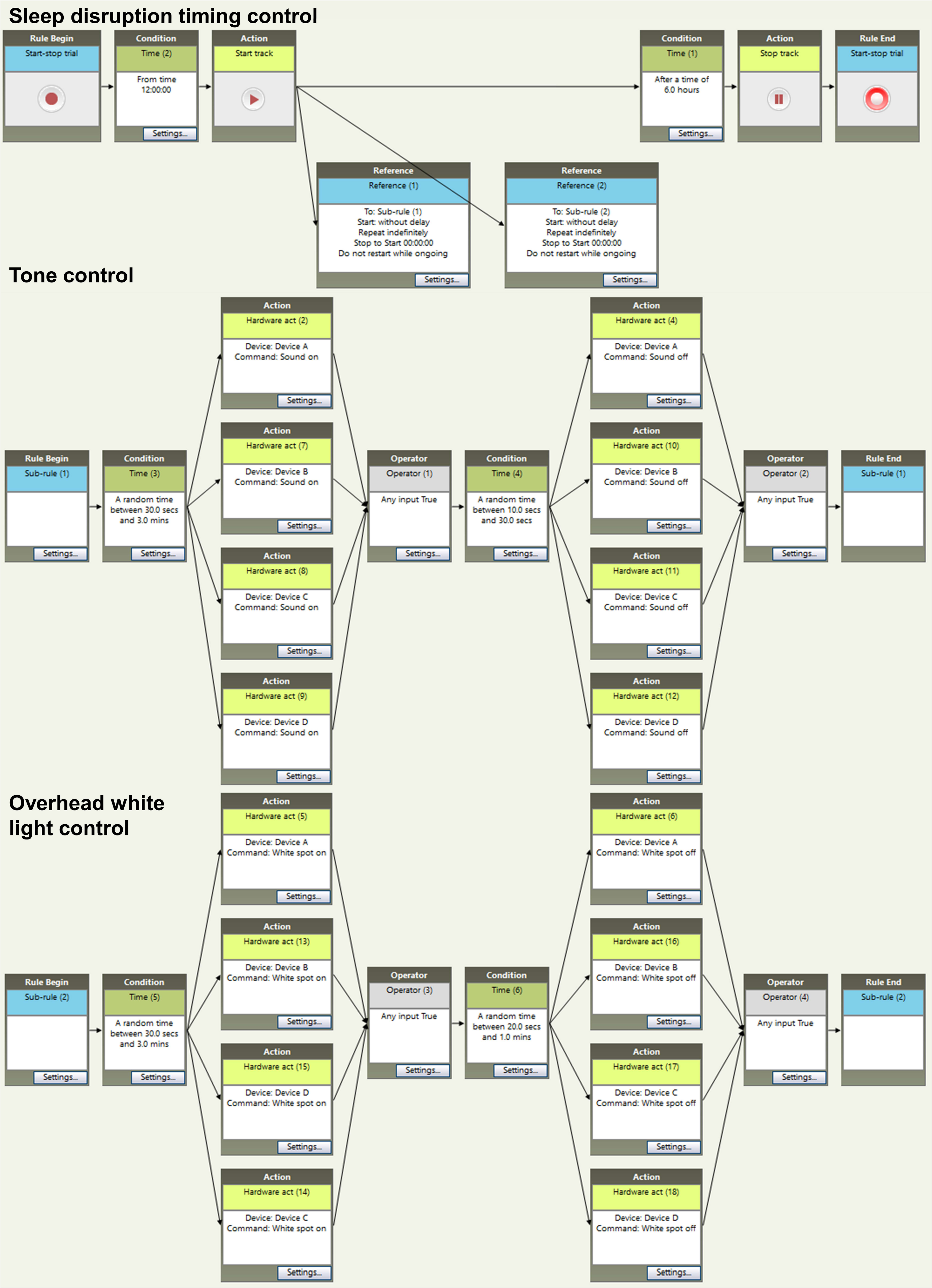
Sleep disruption timing control
Rule Begin
Start-stop trial
Condition
Time (2)
From time 12:00:00
Settings...
Action
Start track
Condition
Time (1)
After a time of 6.0 hours
Settings...
Action
Stop track
Rule End
Start-stop trial
Reference
Reference (1)
To: Sub-rule (1)
Start: without delay
Repeat indefinitely
Stop to Start 00:00:00
Do not restart while ongoing
Settings...
Reference
Reference (2)
To: Sub-rule (2)
Start: without delay
Repeat indefinitely
Stop to Start 00:00:00
Do not restart while ongoing
Settings...
Tone control
Rule Begin
Sub-rule (1)
Condition
Time (3)
A random time between 30.0 secs and 3.0 mins
Action
Hardware act (2)
Device: Device A
Command: Sound on
Action
Hardware act (7)
Device: Device B
Command: Sound on
Action
Hardware act (8)
Device: Device C
Command: Sound on
Action
Hardware act (9)
Device: Device D
Command: Sound on
Operator
Operator (1)
Any input True
Condition
Time (4)
A random time between 10.0 secs and 30.0 secs
Action
Hardware act (4)
Device: Device A
Command: Sound off
Action
Hardware act (10)
Device: Device B
Command: Sound off
Action
Hardware act (11)
Device: Device C
Command: Sound off
Action
Hardware act (12)
Device: Device D
Command: Sound off
Operator
Operator (2)
Any input True
Rule End
Sub-rule (1)
Overhead white light control
Rule Begin
Sub-rule (2)
Condition
Time (5)
A random time between 30.0 secs and 3.0 mins
Action
Hardware act (5)
Device: Device A
Command: White spot on
Action
Hardware act (13)
Device: Device B
Command: White spot on
Action
Hardware act (15)
Device: Device D
Command: White spot on
Action
Hardware act (14)
Device: Device C
Command: White spot on
Operator
Operator (3)
Any input True
Condition
Time (6)
A random time between 20.0 secs and 1.0 mins
Action
Hardware act (6)
Device: Device A
Command: White spot off
Action
Hardware act (16)
Device: Device B
Command: White spot off
Action
Hardware act (17)
Device: Device C
Command: White spot off
Action
Hardware act (18)
Device: Device D
Command: White spot off
Operator
Operator (4)
Any input True
Rule End
Sub-rule (2)

Supplementary Figure 2: Noldus EthoVision trial control settings for sleep disruption in PhenoTypers. Acute sleep disruption was conducted between 12-6pm utilizing a tone and white light in the PhenoTyper top unit. This allowed for a consistent stressor within each mouse cage. The interval between tones and lights, and their duration were procedurally randomized utilizing trial control settings in EthoVision. This figure displays the timing control settings and the sub-rules for both the tone and light. The tone played once every 30s-3min for a duration of 10-30s then repeated indefinitely throughout the sleep disruption period. The light turned on once every 30s-3min for a duration of 20s-1min then repeated indefinitely throughout the sleep disruption period. Each interval and duration were randomized throughout the day. Four hardware actions are documented here because four PhenoTypers were controlled simultaneously. Left to right and arrow direction indicates the order of operations. Images captured from EthoVision XT15.

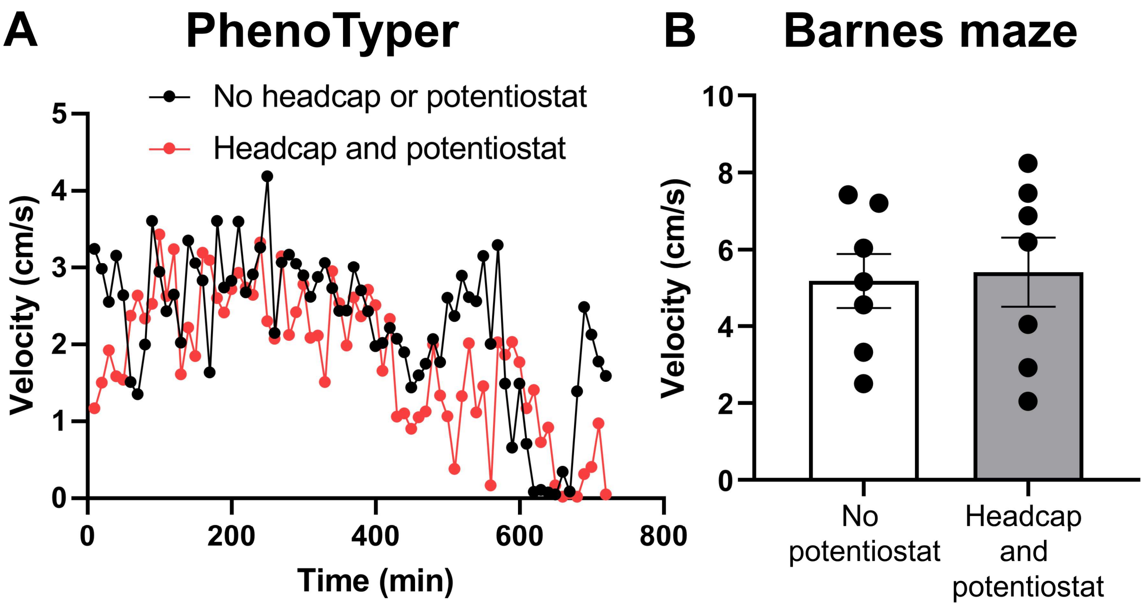

Supplementary Figure 3: Mice quickly habituate to the headcap and potentiostat, and locomotion is not affected. Mice without headcap surgery were compared to mice with headcaps and potentiostat plugged-in (*n*=2/group), in the PhenoTypers. (**A**) During the dark-cycle, no differences in locomotion were detected with the addition of the wireless EEG/EMG unit, indicating normal motor function and exploratory behavior; data are mean at each 10-minute bin in the dark-cycle (720 minutes total). (**B**) In the Barnes maze, we compared velocity of mouse movement in the first minute of the first learning trial (no potentiostat) compared to the first minute of the probe trial (headcap and potentiostat; *n*=7), and observed no significant differences (t=0.17, df=6, P=0.87). Data are mean (**A**) or mean +/- SEM, paired t-test (**B**).

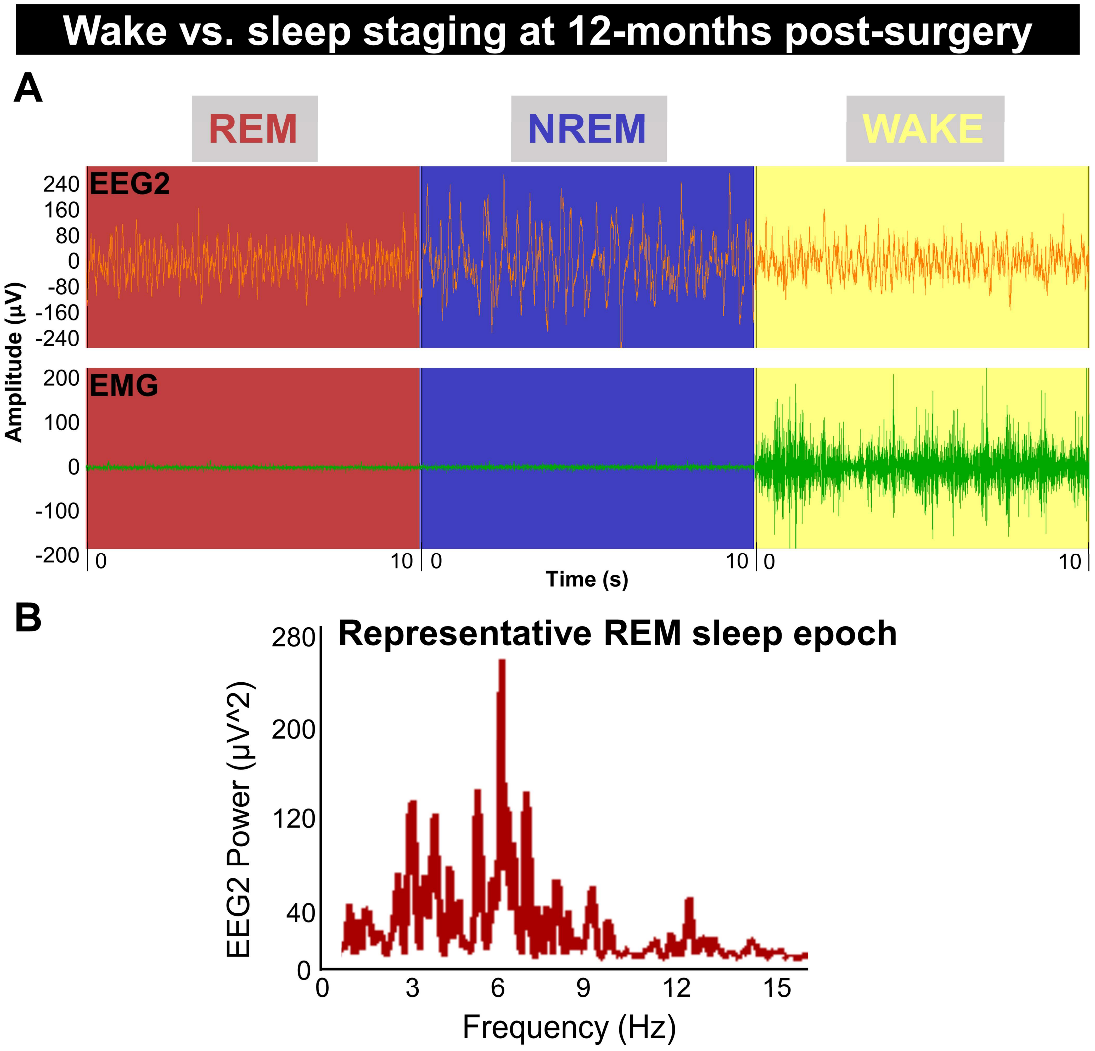

Supplementary Figure 4: Wake and sleep staging at 12-months post-surgery. Supplement to **Figure 3**. Seven mice at 12-months post-surgery were staged for wake, NREM and REM epochs during the light-cycle. (**A**) Representative REM, NREM and wake epochs showing raw EEG2 (anterior) and EMG traces. REM is characterized by low EMG, and mixed-frequency EEG; notably theta is increased, and waves typically appear sharper, and slightly less frequent/higher in amplitude than wake. NREM is characterized by low EMG and delta-dominant EEG: large amplitude, lower frequency oscillations. Wake is characterized by differing, but high EMG activity, and higher frequency/lower amplitude oscillations compared to sleep stages. (**B**) Representative REM sleep epoch demonstrating mixed EEG frequency, and a peak in the theta range (~ 6 Hz). Images captured from Sirenia® Sleep Pro software.

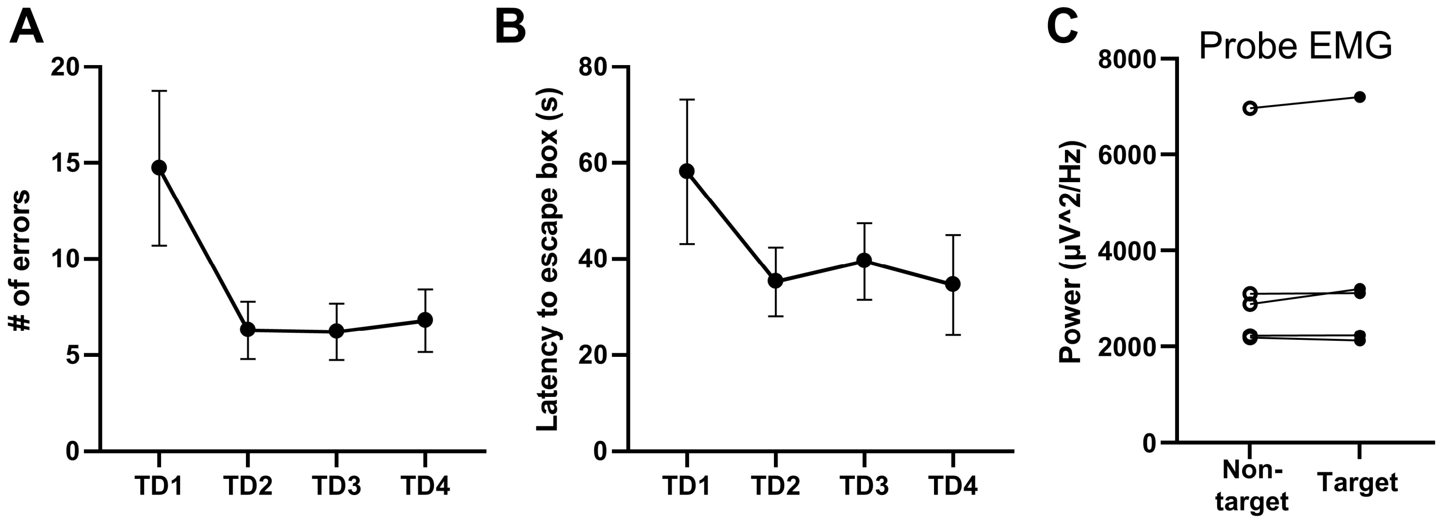

Supplementary Figure 5: Barnes maze learning and EMG during the probe trial. Supplement to **Figure 5**. Mice (*n*=7) with EEG/EMG headcaps were tested in the Barnes maze for their ability to learn the location of the escape box. (**A,B**) Mice demonstrate less errors and quicker latencies to locate the escape box across the 4 learning trial days (TD); errors: trend to effect of trial day (F=4.15, DFn, DFd=1.46, 8.76, P=0.063) and individual subject improvement (F=2.59, DFn, DFd=6, 18, P=0.055); latencies: no significant effect of trial day (F=1.39, DFn, DFd=1.77, 10.62, P=0.29, trend to individual subject improvement (F=2.09, DFn, DFd=6, 18, P=0.11). During the spatial memory probe (3 days later, see **Fig. 5**), potentiostats were plugged-in to acquire EEG/EMG data simultaneous to Barnes maze performance. (**C**) EMG power (50-150 Hz) was generated in 2-second intervals and aligned to position in the Barnes maze for target and non-target quadrants. No significant differences in EMG power were detected by quadrant. Data are mean +/- SEM (**A,B**) or paired before-after comparisons (**C**). Repeated measures one-way ANOVA (**A,B**) and multiple paired t-test with multiple comparisons controlled for with Holm-Šídák method (**C**).
